# Inhibition of EYA family tyrosine phosphatase activity reveals a therapeutic vulnerability and enhances Menin and DOT1L inhibitor efficacy in KMT2A-rearranged leukemia

**DOI:** 10.1186/s40164-025-00717-5

**Published:** 2025-10-22

**Authors:** Lola Badmus, Nicholas J Achille, Shubin Zhang, Xianzhong Ding, Nancy J. Zeleznik-Le

**Affiliations:** 1 Integrative Cell Biology Program, Loyola University Chicago, Maywood, IL USA; 2 Department of Cancer Biology, Loyola University Chicago, 2160 S. First Avenue, Bldg. 112, Rm. 337, Maywood, IL 60153 USA; 3 Pathology and Laboratory Medicine, Loyola University Chicago, Maywood, IL USA

## Abstract

MLL (KMT2A)-rearranged leukemia (MLL-r) is a highly aggressive hematologic malignancy driven by transcriptional dysregulation. Here, we identify EYA family phosphatase activity, particularly EYA1 and EYA3, as key vulnerabilities in MLL-r leukemia. The small molecule benzbromarone (BBR) selectively reduced viability in MLL-r and *EYA*-expressing MLL-nonrearranged (MLL-nr) leukemia cells. Inhibition of EYA PTP activity increased global RNA Pol II CTD Tyr1 phosphorylation, linking aberrant EYA PTP activity in responsive leukemia cells to transcriptional dysregulation. In vivo, BBR treatment significantly prolonged survival and reduced leukemia burden without overt toxicity. Furthermore, BBR synergized with the menin-MLL inhibitor VTP50469 and showed additive effects with the DOT1L inhibitor EPZ5676, the latter of which restored BBR sensitivity in previously BBR-unresponsive cells. These findings establish EYA PTP activity as a therapeutic target in MLL-r leukemia, support the use of *EYA* expression for identifying patients likely to benefit from BBR treatment, and highlight the potential of BBR-based combinations to improve response in this high-risk leukemia subtype.

**Supplementary Information:**

The online version contains supplementary material available at 10.1186/s40164-025-00717-5.

**To the Editor,**

MLL (KMT2A)-rearranged (MLL-r) leukemias are aggressive malignancies driven by MLL fusion oncoproteins, which enhance RNA Pol II elongation and prevent pausing [1]. Although recent targeted therapies have shown promise, durable remission remains elusive for many patients, highlighting the need for novel strategies [2]. We previously identified EYA1, a transcriptional coactivator and protein tyrosine phosphatase (PTP), as a potential therapeutic target in MLL-r leukemia [3]. The EYA family proteins (EYA1–4) function as transcriptional coactivators and PTPs essential in developmental processes. Notably, aberrant expression of EYA1 and EYA3 have each been implicated in cancer progression, including leukemia, through transcriptional regulation and survival signaling [4]. Benzbromarone (BBR), a uricosuric gout drug, was identified as a selective EYA PTP inhibitor [5]. Given our previous finding of EYA1’s critical role in leukemogenesis and the documented involvement of other EYA family members in cancer progression, we hypothesized that targeting multiple EYA family phosphatases with BBR would selectively impair leukemia cell viability. Here, we evaluate the therapeutic potential of EYA family PTP inhibition alone and in combination with menin-MLL and DOT1L inhibitors in MLL-r leukemia.

We treated MLL-AF9 mBM cells and human leukemia cell lines, including MLL-r and MLL-nr subtypes, with increasing concentrations of BBR. Dose-response curves (Fig. 1A) showed significantly reduced viability in MLL-r (MOLM-13, THP-1, SEM), MLL-nr (U-937, Kasumi-1), and MLL-AF9 mBM cells, while MLL-ENL B-ALL lines (HB11;19, KOPN-8) and hCD34+ cells were resistant. IC50 values for responsive lines (Fig. S1A) align with reported human serum levels of BBR in gout patients, supporting the physiological relevance of these findings [6]. We hypothesized that sensitivity may depend on *EYA* expression. Quantitative RT-PCR analysis revealed detectable expression of both *EYA1* and *EYA3*, while *EYA2* and *EYA4* were generally undetectable or minimally expressed (Fig. 1B, Fig. S1B). While resistant cell lines HB11;19 and KOPN-8 expressed low to undetectable levels of both *EYA1* and *EYA3*, responsive leukemia cell lines exhibited varied yet detectable levels of these two phosphatases (Fig. 1B). Given this varied expression, we assessed whether combined *EYA1* and *EYA3* expression might better explain the differential responsiveness. Indeed, linear regression analysis showed a significant inverse correlation between total *EYA1* and *EYA3* expression and BBR IC50 values (Fig. S1C), suggesting that combined expression of these two phosphatases contributes to BBR sensitivity.

**Fig. 1:**
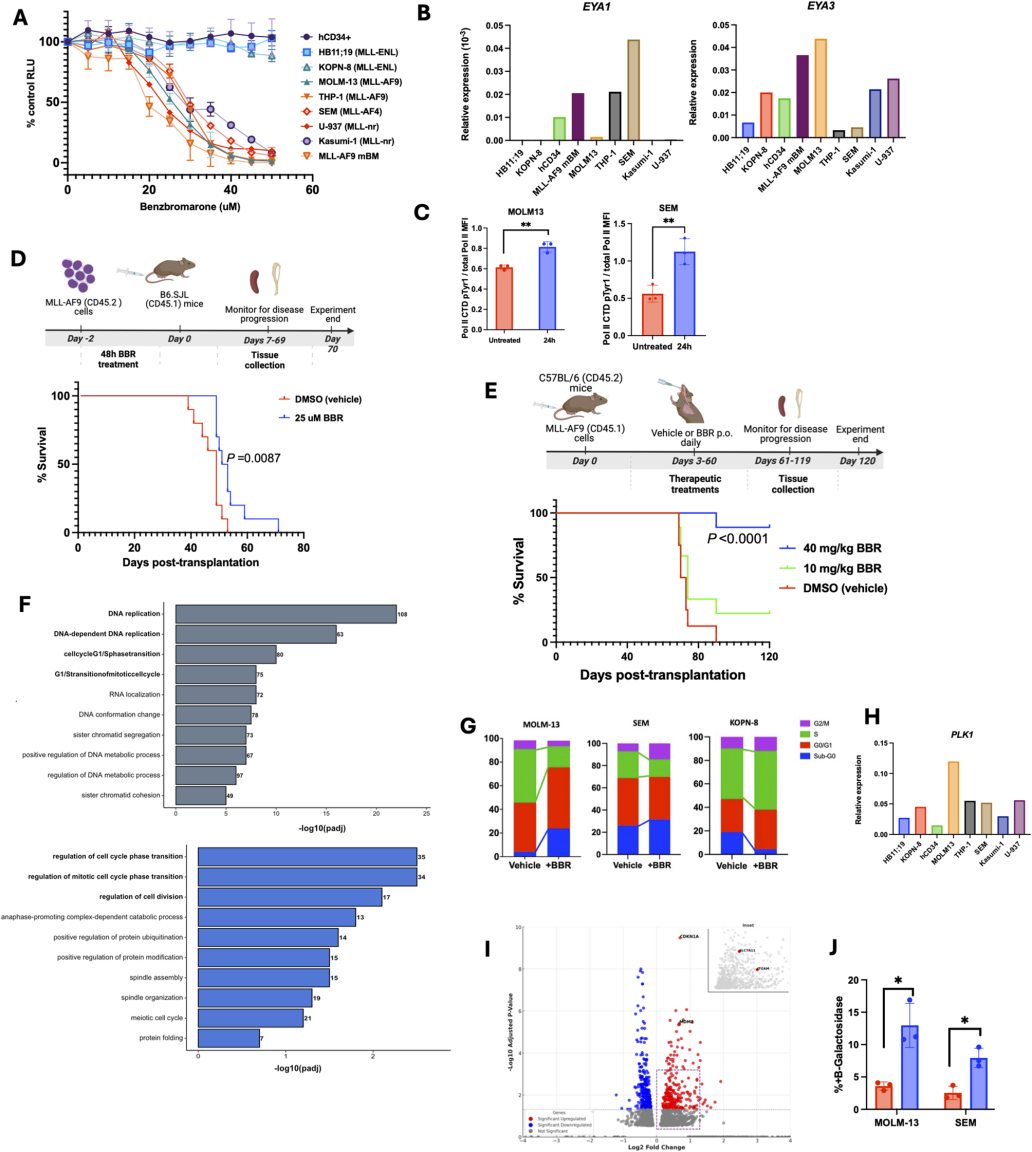
Inhibition of EYA PTP activity reduces leukemia cell viability, alters transcriptional regulation, and delays disease progression. **A** Dose-response curves from Cell Titer Glo 2.0 (CTG 2.0) cell viability assay performed after 72 h vehicle or BBR treatment. Mean ± SD, 3 independent CTG 2.0 assays were performed for each cell type. **B** Quantitative RT-PCR performed in cells with no treatment. Gene expression was normalized to *B2M*. **C** Global RNA Pol II CTD Tyr1 phosphorylation (Pol II CTD pTyr1) in MOLM-13 and SEM cells following 24-h IC50 BBR treatment. Pol II CTD pTyr1 MFI was measured via flow cytometry and was normalized to total Pol II MFI. Mean ± SD, *n* = 3. **D** Schematic diagram showing the transplantation of viable MLL-AF9 mBM cells (CD45.2) that were treated with 30 µM BBR for 48 h into sublethally irradiated (450 cGy) recipient (CD45.1) mice. Kaplan-Meier survival curves of mice that received vehicle or BBR-treated MLL-AF9 mBM cells (*n* = 10). **E** Schematic diagram showing the transplantation of MLL-AF9 mBM cells (CD45.1) into recipient mice, followed by a 10-week (5 days on, 2 days off) oral gavage dosing schedule starting 3 days post-transplantation, using either vehicle (0.5% sodium carboxymethyl cellulose (CMC-Na)), 10 mg/kg, or 40 mg/kg BBR. Kaplan-Meier survival curves of mice that received oral treatment with vehicle, 10 mg/kg, or 40 mg/kg BBR (*n* = 9). **F** Gene Ontology enrichment analysis of biological processes enriched in IC30 BBR-treated MOLM-13 (top) and SEM (bottom) cell lines. Significantly enriched GO terms were identified using gProfiler, with an FDR threshold of < 0.05. **G** Cell cycle analysis of KOPN-8, MOLM-13, and SEM cells following 24-h 30 µM BBR treatment. Representative data are shown (*n* = 2). **H** Quantitative RT-PCR performed in cells with no treatment. Gene expression was normalized to *B2M*. **I** Volcano plot illustrating RNA-seq gene expression analysis of MOLM-13 cells treated at IC30 BBR for 24 h. **J** Flow cytometry analysis of senescence-associated beta-galactosidase in MOLM-13 and SEM cells treated with vehicle or respective IC50 concentrations of BBR for 72 h. Mean ± SD, *n* = 3. *P < 0.05; **P < 0.01 (**C**, **J**)

Previous research showed EYA1 PTP activity influences transcription by dephosphorylating RNA polymerase II (RNA Pol II) at tyrosine 1 (Tyr1) on its C-terminal domain (CTD), a modification enriched during transcriptional elongation and associated with active transcription, promotion of Ser2 phosphorylation, and regulating transcriptional directionality [7–10]. Intracellular staining confirmed that EYA PTP inhibition significantly increased RNA Pol II CTD Tyr1 phosphorylation in responsive MOLM-13 and SEM cells but not in nonresponsive KOPN-8 cells (Fig. 1C, Fig. S1D). Thus, inhibition of EYA1 phosphatase activity may disrupt its aberrant transcriptional regulatory role, contributing to the observed leukemia-specific cytotoxicity. While EYA3 expression correlates with BBR sensitivity as well, its mechanistic role in this context remains to be elucidated.

To test whether EYA PTP inhibition reduces leukemogenic potential, we pretreated MLL-AF9 mBM cells with BBR or vehicle for 48 h before transplanting equal numbers of viable cells into sublethally irradiated mice. Mice receiving BBR-pretreated cells showed a significant survival benefit compared to controls, despite receiving no in vivo treatment (Fig. 1D). We then assessed whether in vivo EYA inhibition could suppress leukemia progression. After confirming tolerability of chronic dosing in healthy mice through monitoring for adverse effects and liver histopathology analysis (Fig. S1E), we transplanted mice with MLL-AF9 mBM cells and administered vehicle, 10 mg/kg, or 40 mg/kg BBR daily via oral gavage. High-dose (40 mg/kg) BBR significantly delayed disease progression, prolonged survival, and significantly reduced leukemia burden, while low-dose (10 mg/kg) treatment significantly reduced leukemia burden but did not impact survival, suggesting a dose-dependent response (Fig. 1E, Figs. S1F, G).

To assess the effect of EYA inhibition on transcriptional regulation, we performed RNA-seq on BBR-treated responsive (MOLM-13 [AML], SEM [ALL]) and nonresponsive (KOPN-8 [ALL]) cells. Gene ontology analysis of responsive cells revealed significant enrichment in cell cycle regulation pathways, particularly G1/S transition (Fig. 1F, Fig. S1H ). Cell cycle analysis confirmed that BBR treatment reduced S-phase and increased sub-G0 fractions in MOLM-13 and SEM cells, suggesting cell cycle arrest and progression toward cell death, whereas KOPN-8 showed resistance (Fig. 1G, Fig. S1I). To explore potential mitotic regulators affected by BBR, we assessed expression of *PLK1*, a known target of EYA1-mediated dephosphorylation at Y445, which is required for proper G2/M progression [11]. qPCR analysis confirmed that all leukemia cell lines and hCD34 + cells express *PLK1* (Fig. 1H). Cell cycle analysis did not reveal a robust G2/M arrest, suggesting that BBR-mediated impairment of the G1/S transition may be the dominant phenotype in this context. Benzbromarone treatment also induced *CDKN1A*, encoding the senescence-associated cell cycle inhibitor p21 (Fig. 1I). Consistent with this, senescence-associated β-galactosidase staining was significantly increased in MOLM-13 and SEM cells following BBR treatment (Fig. 1J, Fig. S1J ). Additionally, EYA PTP inhibition activated multiple cell death pathways, including apoptosis and ferroptosis (Fig. S1K, L).

We investigated whether combining BBR with menin-MLL inhibitor VTP50469 or DOT1L inhibitor EPZ5676 enhances therapeutic response or overcomes resistance. Co-treatment with VTP50469 and BBR significantly reduced viability compared to VTP50469 alone, demonstrating strong synergism in MOLM-13 cells and an additive effect in SEM cells (Fig. 2 A, Fig. S2A). Additionally, MOLM-13 cells pretreated with EPZ5676 showed enhanced sensitivity to subsequent BBR treatment, indicating an additive effect (Fig. S2B). Pretreatment of the nonresponsive KOPN-8 line with EPZ5676 induced cellular senescence and sensitized cells to BBR, significantly reducing viability compared to either agent alone. There was no consistent change in EYA1 expression and a decrease in EYA3 expression following combination treatment (Fig. 2B, Fig. S2C, D). This reduction in EYA3 suggests that the observed phenotype may result from multiple factors, which will require further study to elucidate. Notably, combination treatment had no impact on viability in hCD34 + cells, further supporting the leukemia-specific activity of BBR (Fig. S2E).

**Fig. 2:**
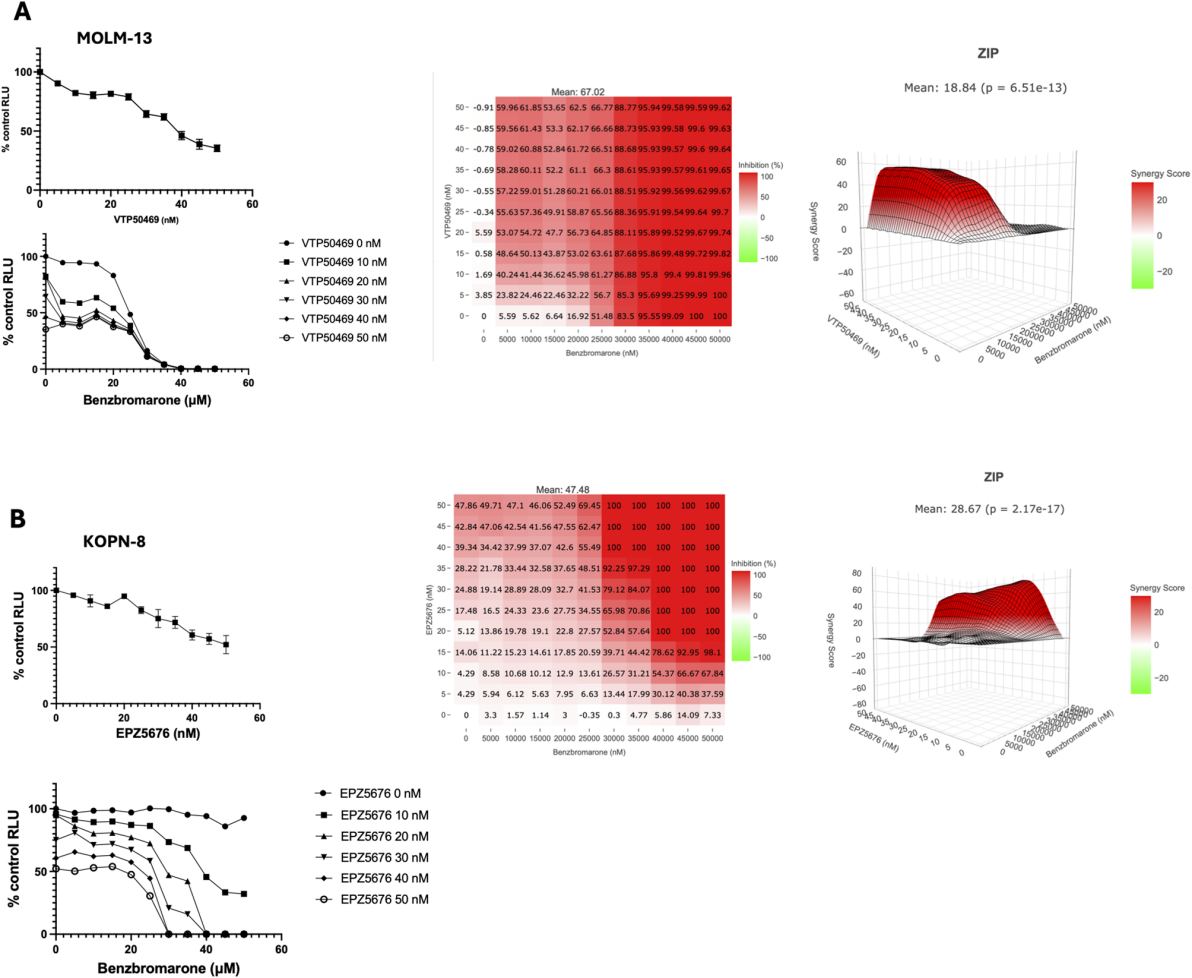
Combined treatment with Menin inhibitor and BBR synergistically reduces cell viability in responsive cell line and sensitizes nonresponsive cell line to BBR. **A** CTG 2.0 assay of MOLM-13 cells treated for 96 h with BBR and VTP50469, alone or in combination, using a full titration matrix to assess dose–response and synergy. Mean ± SD, *n* = 3. Synergy score was calculated using % viability data with the ZIP method via SynergyFinder+. Scores > 10 indicate synergism, scores between − 10 and 10 indicate an additive effect, and scores < − 10 indicate antagonism. **B** CTG 2.0 assay of KOPN-8 cells treated for 96 h with BBR and EPZ5676, alone or in combination, using a full titration matrix to assess dose–response and synergy. Mean ± SD, *n* = 3. Synergy score determined as indicated in **A**

Prior studies implicated *EYA1* expression in MLL-r leukemogenesis [3, 12]. Here, we demonstrate that PTP activity inhibition of EYA family members, particularly EYA1 and EYA3, is sufficient to induce significant antileukemic effects, thereby validating EYA PTP activity as a novel therapeutic vulnerability in MLL-r leukemia. Additionally, *EYA1* and *EYA3* expression can be a criterion for patient stratification. Mechanistically, increased RNA Pol II CTD Tyr1 phosphorylation following BBR treatment suggests disrupted transcriptional regulation as a mechanism of action for aberrantly expressed EYA in MLL-r leukemia. Furthermore, BBR treatment exerts its antileukemic effects in part by inducing cell cycle arrest and cellular senescence. In vivo, BBR significantly reduced leukemia burden, improved survival, without hepatotoxicity at effective doses, addressing previous debated safety concerns [13]. Although BBR has been reported to inhibit additional targets beyond the EYA family [5, 14–16], our analysis of DepMap CRISPR dependency and expression datasets indicates that these proteins are not expressed or essential in AML or B-ALL cell lines (Supplemental Table 1). This suggests the observed phenotypes are not driven by reported off-targets but rather by EYA family phosphatase inhibition.

We identified BBR’s synergism with menin inhibitor VTP50469 and additive effects with DOT1L inhibitor EPZ5676. These findings align with Miao et al., who also showed enhanced efficacy of combination treatments in leukemia models [17]. DOT1L inhibition sensitized BBR-resistant KOPN-8 cells to subsequent BBR treatment, suggesting potential for expanding BBR responsiveness.

Our study establishes EYA family PTP activity as a previously unrecognized vulnerability in MLL-r leukemia and supports repurposing BBR as part of combination therapies to improve treatment outcomes.

## Supplementary Information

Below is the link to the electronic supplementary material.

Supplementary Material 1.

## Data Availability

All raw data supporting the findings of this study are available from the corresponding author upon reasonable request. RNA sequencing data have been deposited in the NCBI Sequence Read Archive (SRA) under the accession number PRJNA1233325.
