# Supplementary material for: Inhibition of EYA family tyrosine phosphatase activity reveals a therapeutic vulnerability and enhances Menin and DOT1L inhibitor efficacy in KMT2A-rearranged leukemia
Source: Exp Hematol Oncol. 2025 Oct 22;14:126. doi: 10.1186/s40164-025-00717-5 (PMC12548103; doi:10.1186/s40164-025-00717-5)
Supplement: Supplementary file 1 — Supplementary Material 1. [file 40164_2025_717_MOESM1_ESM.pdf]

A

Leukemia cells	IC50 value (μM)
MLL-AF9 mBM	21.4
U-937 (MLL-nr)	23.5
MOLM-13 (MLL-AF9)	24.3
THP-1 (MLL-AF9)	27.1
SEM (MLL-AF4)	29.1
Kasumi-1 (AML-ETO)	30.1
HB11;19 (MLL-ENL)	No response
KOPN-8 (MLL-ENL)	No response
Normal human CD34+ bone marrow cells	No response

B

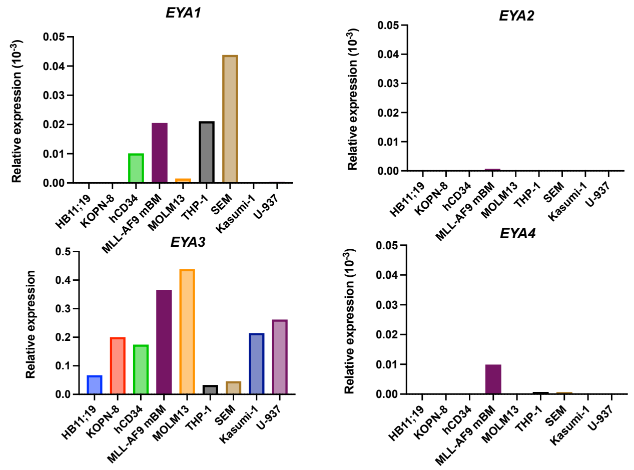

C

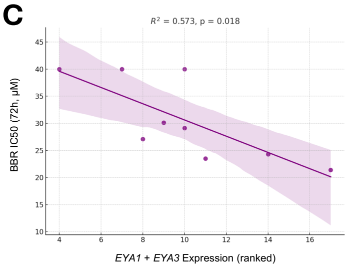

D

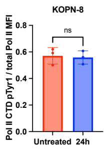

E

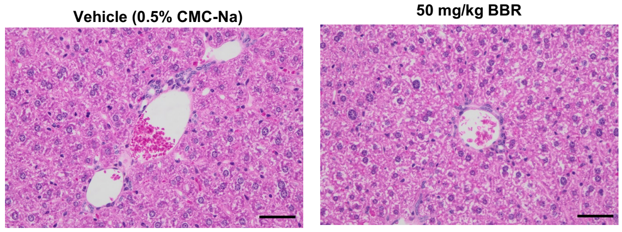

F

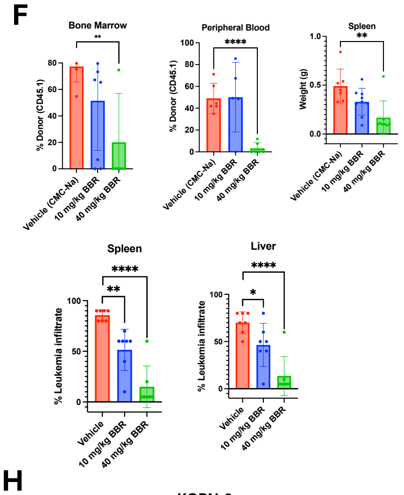

G

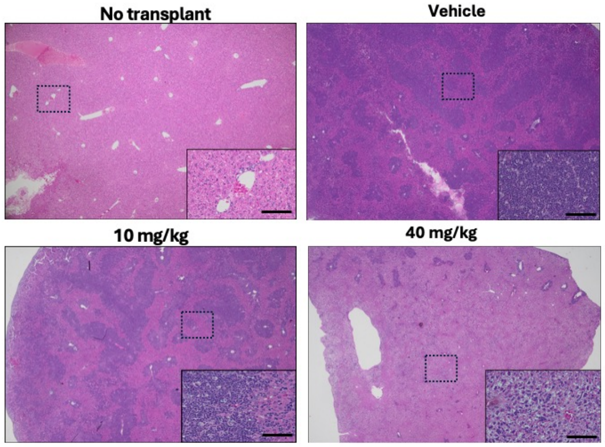

H

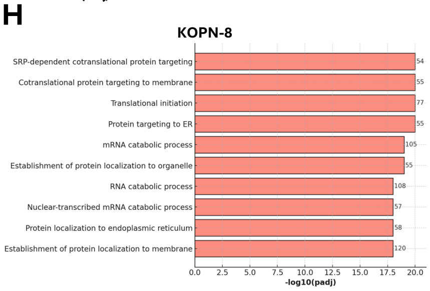

I

	$<G_0$		G_0/G_1		S		G_2/M	
	DMSO	BBR	DMSO	BBR	DMSO	BBR	DMSO	BBR
MOLM-13	3.96	23.70	41.70	51.70	45.40	18.00	7.27	4.57
	26.2	58.5	45.7	28.5	21.9	10.1	6.16	2.88
SEM	25.90	31.40	42.80	38.40	24.20	15.90	7.10	14.30
	18.9	29.3	28.1	24.5	43.2	37.2	9.62	8.78
KOPN-8	18.90	4.30	28.10	33.70	43.20	50.20	9.80	11.80
	6.70	4.60	31.30	33.00	50.30	50.10	11.24	12.10

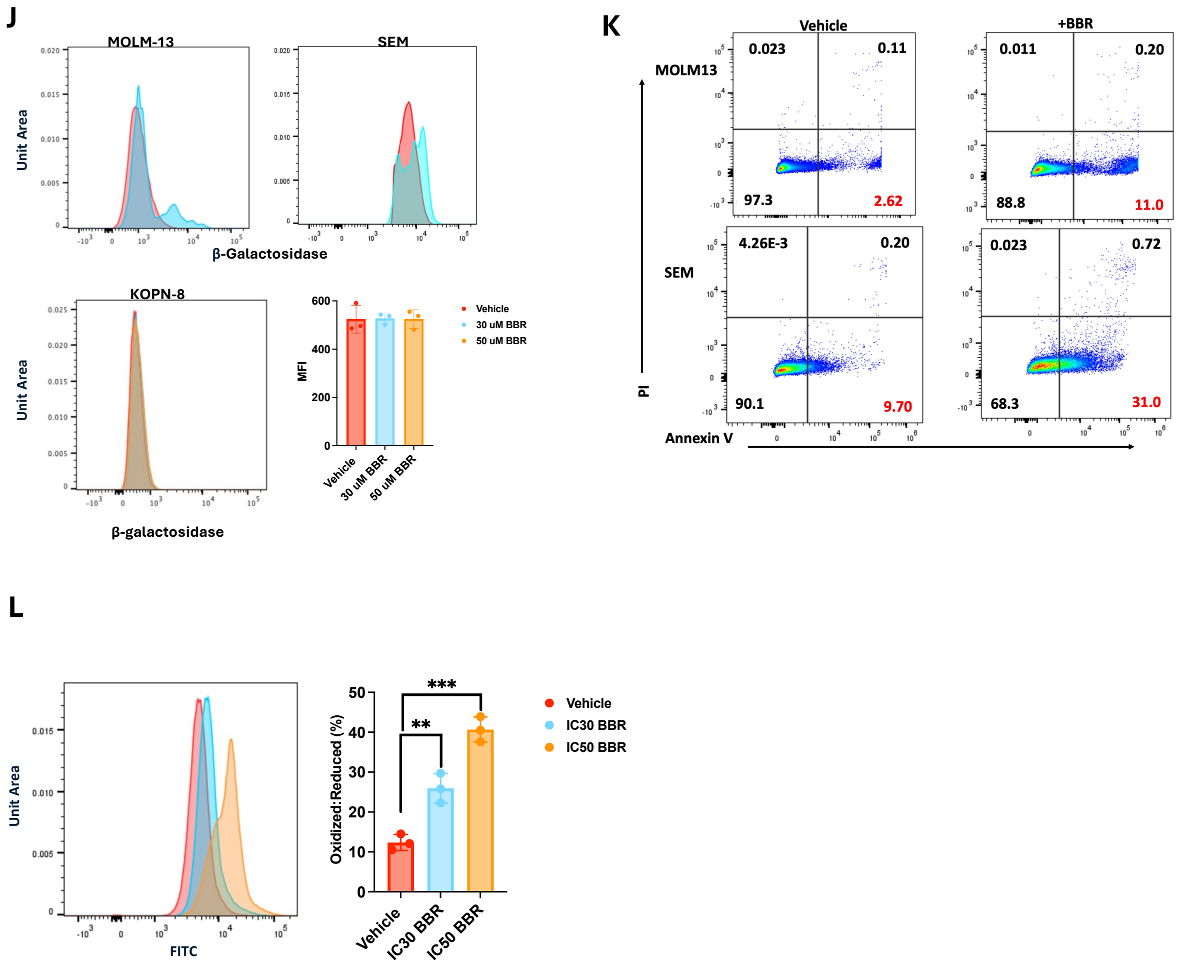

Figure S1. EYA PTP inhibition reduces leukemia cell viability, alters Pol II phosphorylation, and induces diverse cell death responses.

(A) IC50 values were calculated with Excel add-in ED50V10 using CTG 2.0 cell viability data. Mean of 3 independent experiments shown in table. **(B)** Quantitative RT-PCR performed in cells with no treatment. Gene expression was normalized to *B2M*. **(C)** Linear regression analysis of 72h BBR IC50 values against *EYA1* and *EYA3* expression in treated cells. Expression values were ranked and rankings were combined and plotted against IC50 values, with values plotted as the mean of biological triplicates. **(D)** Quantification of RNA Pol II CTD phospho-Tyr1 MFI levels in DMSO- and 41.6 μM BBR-treated (24h) KOPN-8 cells. RNA Pol II pTyr1 MFI is normalized to total Pol II MFI. Data are presented as mean ± SD (n = 3 biological replicates). **(E)** H&E-stained liver sections from mice treated with vehicle (0.5% CMC-Na) or 50 mg/kg BBR daily (5 days on, 2 days off) for 8 weeks show normal liver morphology across all cohorts. Representative images are shown. Scale bar = 50 μm. **(F)** Flow cytometry analysis of percent CD45.1+ (donor) cells in the bone marrow and

peripheral blood and spleen weights of mice at time of sacrifice. Percent leukemia infiltration in spleen and liver of mice at the time of sacrifice as determined by blinded histopathological analysis. **(G)** H&E-stained liver sections from mice treated with no transplant, vehicle, 10 mg/kg, and 40 mg/kg of BBR. Leukemia infiltration was quantified by a pathologist with blinded samples. Representative images show liver morphology across cohorts. Insets provide a closer view of cellular architecture within each section. Scale bar: 100 μm. Mean ± SD. *P < 0.05; **P < 0.01; ***P < 0.001; ****P < 0.0001. **(H)** GO analysis of biological processes in KOPN-8 cells highlights the differences in enriched pathways compared to responsive cell lines. DMSO- versus 20.2 μM BBR-treated samples were analyzed (n = 3 biological replicates per group). **(I)** Table showing cell cycle phase distributions (sub-G0, G0/G1, S, and G2/M) in KOPN-8, MOLM-13, and SEM cells treated with DMSO or BBR at IC50 concentrations (41.6 μM for KOPN-8) for 24h (*n* = 2 biological replicates per group). **(J)** Representative histograms of senescence-associated beta-galactosidase in MOLM-13 and SEM cells treated with vehicle or respective IC50 concentrations of BBR for 72 hours. Senescence analysis of 72h treatment of KOPN-8 cells with DMSO, 30, or 50 μM BBR. Representative histogram and MFI quantification. Data are presented as mean ± SD (n = 3 biological replicates). **(K)** Flow cytometry analysis of Annexin V/PI staining in MOLM-13 and SEM cells treated with BBR at their 12-hour IC50 concentrations. Plot represents distribution of cells in early apoptosis (Annexin V+/PI-), late apoptosis (Annexin V+/PI+), necrosis (Annexin V-/PI+), and live cells (Annexin V-/PI-) following treatment. Representative plot, n = 3. **(L)** Flow cytometry analysis of BODIPY 581/591 C11 lipid peroxidation sensor in vehicle-treated and 24-hour BBR-treated MOLM-13 cells. Representative histogram, mean ± SD, n = 3.

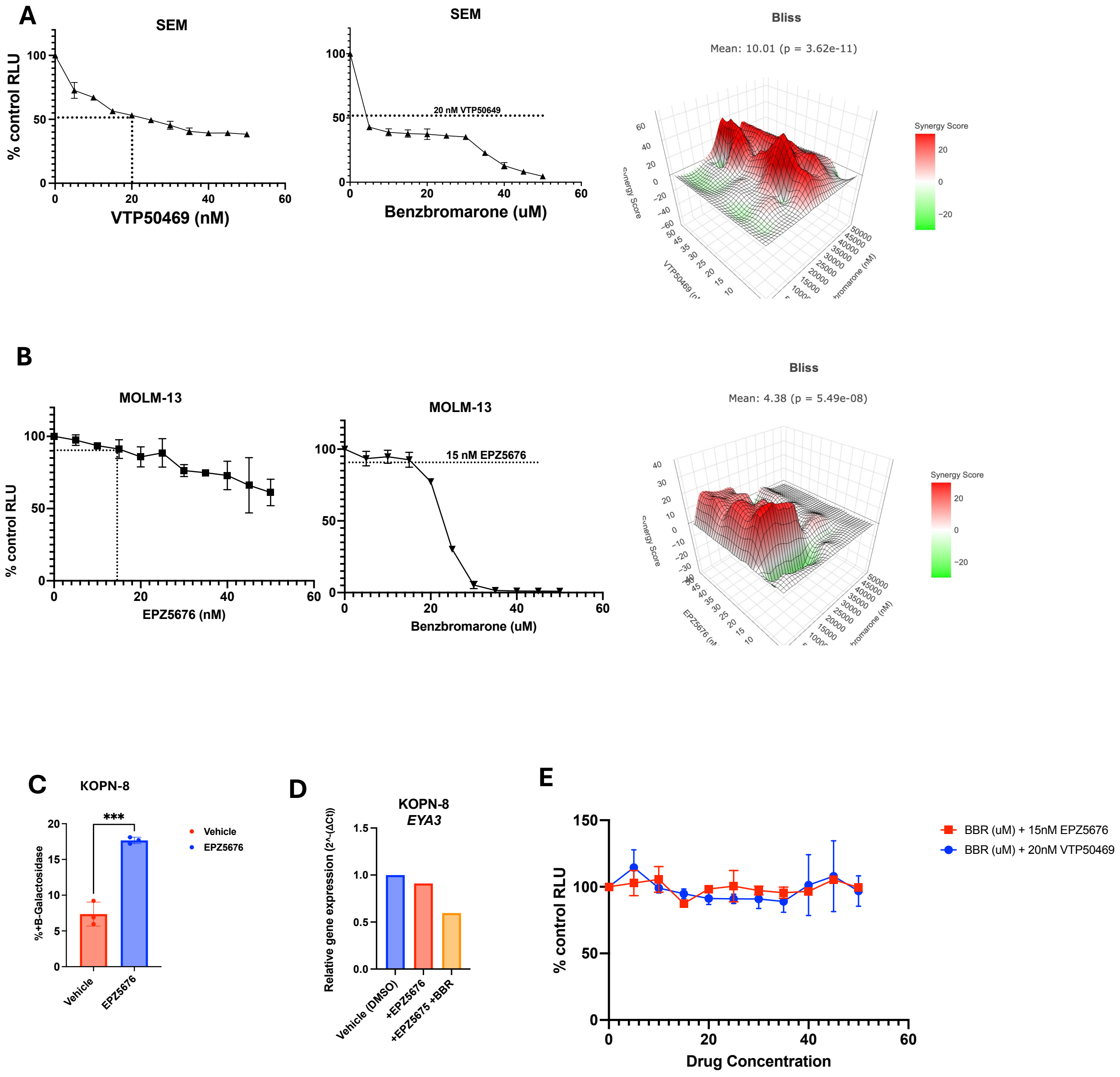

Figure S2. EYA1 PTP inhibition enhances targeted therapy response and DOT1L inhibition induces senescence in BBR-resistant leukemia cells.

(A) CTG 2.0 assay performed on SEM cells treated with increasing concentrations of VTP50469 and with 20 nM VTP50469 combined with increasing concentrations of BBR for 96 hours. Mean ± SD, n = 3. Synergy score was calculated using % viability data with the Bliss method via SynergyFinder+. Scores >10 indicate synergism, scores between -10 and 10 indicate an additive effect, and scores <-10 indicate antagonism. **(B)** CTG 2.0 assay performed on MOLM-13 cells treated with EPZ5676 for 14 days or pretreated with 15 nM

EPZ5676 for 14 days, followed by treatment with increasing concentrations of BBR for 72 hours. Mean ± SD, n = 3. Synergy score determined as indicated in **A.** **(C)** Quantification of the induction of senescence-associated β-galactosidase in KOPN-8 cells treated with DMSO or 15 nM Pinometostat (EPZ5676) for 2 weeks. Data are presented as mean ± SD (n = 3 biological replicates). **(D)** Quantitative RT-PCR performed KOPN-8 cells treated with EPZ5676 for 14 days or EPZ5676 for 14 days followed by BBR for 72h. Gene expression was normalized to *B2M*. **(E)** CTG 2.0 assay performed on hCD34+ cells treated with increasing concentrations of BBR with 15nM EPZ5676 or 20 nM VTP50469. Mean ± SD, n = 3.

Gene	Disease	N cell lines	Median Dependency	Mean Dependency	Median Expression (TPM)
CYP2C9	Acute Myeloid Leukemia	30	0.102	0.104	0.0
CYP2C9	B-Cell Acute Lymphoblastic Leukemia	12	0.034	0.053	0.0
CYP3A4	Acute Myeloid Leukemia	30	0.03	0.038	0.011
CYP3A4	B-Cell Acute Lymphoblastic Leukemia	12	0.032	0.058	0.024
PTPN1 (PTP1B)	Acute Myeloid Leukemia	30	-0.01	0.044	4.616
PTPN1 (PTP1B)	B-Cell Acute Lymphoblastic Leukemia	12	-0.09	-0.06	4.794
SLC22A12 (URAT1)	Acute Myeloid Leukemia	30	-0.007	0.013	0.0
SLC22A12 (URAT1)	B-Cell Acute Lymphoblastic Leukemia	12	0.049	0.048	0.0
SLC22A6 (OAT1)	Acute Myeloid Leukemia	30	0.021	0.014	0.0
SLC22A6 (OAT1)	B-Cell Acute Lymphoblastic Leukemia	12	-0.022	-0.032	0.0
SLC22A8 (OAT3)	Acute Myeloid Leukemia	30	-0.049	-0.045	0.0
SLC22A8 (OAT3)	B-Cell Acute Lymphoblastic Leukemia	12	-0.081	-0.078	0.0

Supplementary Table 1. DepMap Public 25Q3 (Chronos) CRISPR analysis of reported BBR off-targets (*URAT1/SLC22A12, OAT1/SLC22A6, OAT3/SLC22A8, CYP2C9, CYP3A4, and PTPN1/PTP1B*) in AML and B-ALL cell lines. Shown are the number of lines, median and mean dependency scores, and median expression (TPM).

Primer	Sequence (5’ to 3’)	Source
B2M		IDT
Probe	/5HEX/CCTGCCGTG/ZEN/TGAACCATGTGACT/3IABkFQ/	
Primer 1	GGACTGGTCTTTCTATCTCTTGT	
Primer 2	ACCTCCATGATGCGCTTAC	
B2m		IDT
Probe	/5HEX/CCGGAGAAT/ZEN/GGGAAGCCGAACATAC/3IABkFQ/	
Primer 1	TGGTCTTTCTGTGCTTGTTC	
Primer 2	GGGTGGAACGTGTTACGTAG	
Eya1		IDT
Probe	/56-FAM/ACAGACCCC/ZEN/ACAGCAGAGTACAGTA/3IABkFQ/	
Primer 1	CTTCCCATCTGAACCTCGAC	
Primer 2	AATGCCACTTACCAACTCCAG	
EYA1		IDT
Probe	/56-FAM/CTAGCCAC/ZEN/GTACCCACAGCCA/3IABkFQ/	
Primer 1	CACAGGAATGCAACAAGCTAC	
Primer 2	TGTGACAATCCACCTTCAGTC	
EYA2		IDT
Probe	/56-FAM/AGTTCTACC/ZEN/ATTTCCTTGTACCGCGGC/3IABkFQ/	

Primer 1	AACGGGAGAGACAGCAAC	
Primer 2	TGAGGCTGGGTGAGATCA	
Eya2		IDT
Probe	/56-FAM/CACCACCCT/ZEN/ATACAGCGGTACCCAA/3IABkFQ/	
Primer 1	AGTACAGGCACAGGCATTCAG	
Primer 2	GAGTGATTCAAACTGTCTTCTGTC	
EYA3		IDT
Probe	/56-FAM/ACTGGATTG/ZEN/CAAGTCTCTCTCAGCAG/3IABkFQ/	
Primer 1	ACTGATCTCCATGTGTCCTGTTG	
Primer 2	GTTGCTCTGGTAAATCTTGCTC	
Eya3		IDT
Probe	/56-FAM/CCAGATGGCC/ZEN/AGTGATGAGAAGGAGGCCT/3IABkFQ/	
Primer 1	GAAGAGCAAGACCTACCAGAG	
Primer 2	CTGACATGCTGAGATTTGACG	
EYA4		IDT
Probe	/56-FAM/TTCCCGGAAG/ZEN/TGTCTGTCCAGC/3IABkFQ/	
Primer 1	CTTAATTACGGGACGGCACTGAAC	
Primer 2	CCACTCCCAAGTAGAAGCAG	
Eya4		IDT

Probe	/56-FAM/TGAGCAATC/ZEN/TGTAAAGAAAAACGTGCCC/3IABkFQ/	
Primer 1	CAGTGGCAAGAGAAGTTAGAGA	
Primer 2	CTGGAATTCTGAGGTTCCGAA	
PLK1		IDT
Probe	/56-FAM/AGCTCCAAC/ZEN/ACCACGGAACACGA/3IABkFQ/	
Primer 1	CGTAGGATTCCACCAGGCTTT	
Primer 2	GCTTTCCTCCTCTTGTGCAG	
Polr2a		IDT
Probe	/5HEX/CCACCACCT/ZEN/CTTCCTCCTCTTGC/3IABkFQ/	
Primer 1	GGTCCTTCGAATCCGCATC	
Primer 2	CAGGGTCATATCTGTCAGCATG	

Supplementary Table 2. Primers Used for qRT-PCR

Methods

Small-Molecule Inhibitors

Pinometostat (EPZ5676, #S7062), VTP50469 (#S8934), and BBR (#S4221) were purchased from Selleckchem (Houston, TX). Aliquots in 100% DMSO (Sigma-Aldrich, #D2650, Milwaukee, WI) were stored at −80 °C to prevent multiple freeze-thaw cycles.

Cell lines and cell culture

Leukemia cell lines MOLM-13, SEM, THP-1, KOPN-8, Kasumi-1, and U-937 were purchased from ATCC (Manassas, VA) or DMSZ (Braunschweig-Süd, Germany). HB11;19 cells were kindly provided by Dr. Sarah K. Tasian at Children's Hospital of Philadelphia and Dr. Patrick Brown at Johns Hopkins. Healthy donor $CD34^+$ peripheral blood stem cells were obtained as fully deidentified apheresis products from Key Biologics (Memphis, TN), now Charles River Laboratories (Wilmington, MA). MOLM-13, SEM, THP-1, KOPN-8, Kasumi-1, HL-60, and HB11;19 cells were cultured in RPMI-1640 medium (Fisher Scientific, #MT10104CV, Waltham, MA) supplemented with 10% fetal bovine serum (FBS) and 1% penicillin/streptomycin (P/S). Human $CD34^+$ cells were cultured in StemSpan SFEM II medium (Stem Cell Technologies, #09655, Cambridge, MA) supplemented with 50 ng/mL TPO (#300-18), FLT3 (#300-19), and IL-6 (PeproTech, #200-07, Cranbury, NJ). MLL-AF9-transformed mouse bone marrow cells were cultured in RPMI-1640 medium with 10% FBS, 1% P/S, and 10 ng/mL IL-3 (#213-13), IL-6 (#216-16), and SCF (PeproTech, #250-03). The cell lines used in these studies were authenticated by the Tumor Engineering and Phenotyping Facility at the University of Illinois at Champaign-Urbana via STR profiling.

Cell Viability

Viability was assessed using CellTiter-Glo 2.0 (Promega #G9242) on a BioTek Synergy H1 microplate reader. IC50 values were calculated with ED50V10 (Excel). Hema-3 stain (Thermo #122911) was applied post-cytospin. Cells received 96 h co-treatment (BBR + VTP50469) or 14-day EPZ5676 pretreatment followed by 72 h BBR. Synergy was analyzed

via SynergyFinder+ (ZIP method [21]). Synergy analysis was performed using SynergyFinder+ (https://synergyfinder.org) with scores calculated via the ZIP and Bliss methods [1-3].

RNA Isolation and Quantitative RT-PCR

RNA was isolated with TRI Reagent (Sigma #T9424), reverse transcribed (High-Capacity cDNA Kit, Applied Biosystems #4368813), and amplified using TaqMan probes (*EYA1/Eya1*, *CDKN1A*; IDT) with TaqMan Fast Advanced Master Mix (#4444557). Expression was normalized to *B2M/Pol2ra* and analyzed via $2^{-\Delta\Delta Ct}$ [4].

RNAseq analysis

RNA from two BBR-responsive and one nonresponsive line (DMSO vs. IC30 or 20.2 μM BBR, 24h; n=3 biological replicates) was isolated using the RNeasy Mini Kit (Qiagen, #74104, Germantown, MD). RNA sequencing was performed by Novogene (Sacramento, CA). RNA concentration and purity were assessed with the Agilent 5400, and RNA integrity was confirmed using a Bioanalyzer. Samples with RNA Integrity Numbers (RIN) > 8 were used for RNA sequencing. mRNA was isolated from total RNA enriched using poly-T oligo-attached magnetic beads, fragmented, and used for cDNA library preparation with directional strand specificity. Libraries were sequenced using Illumina NovaSeq 6000, generating paired-end 150 bp reads with an average depth of 30 million reads per sample. Raw reads were quality-checked and trimmed using fastp, then aligned to the GRCh38 human genome using HISAT2 v2.0.5. Gene expression levels were quantified with featureCounts, and differential

expression analysis was performed using DESeq2. Genes with an adjusted p-value ≤ 0.05 were considered significantly differentially expressed. Pathway enrichment analyses (GO, KEGG, and Reactome) were conducted using the clusterProfiler R package, and gene set enrichment analysis (GSEA) was performed using the Broad Institute's GSEA tool.

In vivo transplantation

Studies involving mice were approved by Loyola University's Institutional Animal Care and Use Committee, according to standards set forth in the NIH Guidelines. B6.SJL (#002014) and C57BL/6 (#000664) mice were purchased from The Jackson Laboratory (Bar Harbor, ME). Bone marrow cells from C57BL/6 (CD45.2) and B6.SJL (CD45.1) were retrovirally transduced as previously described [5]. MLL-AF9 bone marrow cells (CD45.2) were treated with DMSO or BBR for 48 hours. Viable cells were counted via trypan blue exclusion, and 1.25×10^5 viable cells were transplanted into 8-week-old sublethally irradiated (450 cGy, Radsource RS-2000) B6.SJL female mice via tail vein injection.

Mice were prophylactically treated with Baytril (Enrofloxacin, MilliporeSigma, #17849, Burlington, MA) at 0.1 mg/mL in water for 14 days post-irradiation. 1.25×10^5 MLL-AF9 bone marrow cells (CD45.1) were transplanted into 6-week-old female C57BL/6 mice via tail vein injection. Mice were monitored for leukemia development, and bone marrow was harvested from moribund animals. Bone marrow cells were analyzed for CD45.1$^+$ leukemic populations, and samples with >90% CD45.1$^+$ cells were expanded in MethoCult M3234 (Stem Cell Technologies, #03234, Cambridge, MA) supplemented with 10 ng/mL IL-3, IL-6,

and SCF (PeproTech). Expanded MLL-AF9 CD45.1^{+} cells were used in subsequent transplant studies.

1×10^{6} MLL-AF9 CD45.1 cells were transplanted into C57BL/6 mice, followed by vehicle (0.5% CMC-Na) or BBR (10/40 mg/kg) on a 5-days-on/2-off schedule. Mice were monitored daily for leukemia development by observing significant weight loss, reduction in activity levels, and changes in ear and foot color. Bone marrow, spleens, livers, and peripheral blood were collected at the time of sacrifice. CD45.1/CD45.2 staining was used to determine leukemia cell contribution. Liver and spleen tissue samples were paraffin-embedded, sectioned, and H&E stained (VitroVivo Biotech, Rockville, MD). H&E-stained tissues were blinded and scored by a pathologist for extent of leukemia infiltration.

Flow cytometry analysis

Cells were stained with antibodies against CD45.1 (eBiosciences, #12-0453-82, RRID: AB_465675), CD45.2 (eBiosciences, #11-0454-85, RRID: AB_465062), RNA Pol II CTD phospho-Tyr1 (Active Motif, #61383, RRID: AB_2793613), and RNA Pol II (Active Motif, #39097, RRID: AB_2732926). Additional staining was performed using the Annexin V/Dead Cell Apoptosis Kit (Thermo Fisher Scientific, #V13242), BODIPY 581/591 C11 lipid peroxidation sensor (Thermo Fisher Scientific, #D3861), and the Cell Meter Cellular Senescence Activity Assay Kit (AAT Bioquest, #23005, Pleasanton, CA), per the manufacturer's instructions.

DepMap analysis

Gene expression (TPM) and dependency scores (Chronos) were obtained directly from the DepMap Public 25Q3 release (https://depmap.org/portal/). Reported BBR off-targets (URAT1/SLC22A12, OAT1/SLC22A6, OAT3/SLC22A8, CYP2C9, CYP3A4, and PTPN1/PTP1B) were queried, and summary data for AML and B-ALL cell lines were exported from the portal and presented in Supplemental Table 1.

1. Ianevski, A., et al., *SynergyFinder: a web application for analyzing drug combination dose-response matrix data*. Bioinformatics, 2017. **33**(15): p. 2413-2415.
2. Yadav, B., et al., *Searching for Drug Synergy in Complex Dose-Response Landscapes Using an Interaction Potency Model*. Comput Struct Biotechnol J, 2015. **13**: p. 504-13.
3. Zheng, S., et al., *SynergyFinder Plus: Toward Better Interpretation and Annotation of Drug Combination Screening Datasets*. Genomics Proteomics Bioinformatics, 2022. **20**(3): p. 587-596.
4. Livak, K.J. and T.D. Schmittgen, *Analysis of relative gene expression data using real-time quantitative PCR and the 2(-Delta Delta C(T)) Method*. Methods, 2001. **25**(4): p. 402-8.
5. Cierpicki, T., et al., *Structure of the MLL CXXC domain-DNA complex and its functional role in MLL-AF9 leukemia*. Nat Struct Mol Biol, 2010. **17**(1): p. 62-8.
